# Endoscopic removal of a retained esophageal stent using the stent-in-stent technique

**DOI:** 10.1016/j.vgie.2022.08.019

**Published:** 2022-10-07

**Authors:** Jose Antonio Navarro Almario, Shruti Mony, Danse Bi, Olaya Brewer-Guttierez

**Affiliations:** Division of Gastroenterology and Hepatology, Johns Hopkins Hospital, Baltimore, Maryland

**Keywords:** SEMS, self-expanding metal stent, SEPS, self-expanding plastic stent

## Abstract

Video 1 Stent-in-stent technique for removal of retained esophageal self-expanding metal stent.

Stent-in-stent technique for removal of retained esophageal self-expanding metal stent.

The use of esophageal self-expanding metal stents (SEMSs) has been shown to be advantageous in the setting of malignant dysphagia; they are commonly used for esophageal obstruction and fistulas. Their use in benign esophageal disease is not routine; however, given the risk of adverse events, including tissue ingrowth at the uncovered portions, migration, and bleeding.^1^ Roux-en-Y gastric bypass is the most common bariatric procedure performed worldwide, and stricturing of the gastrojejunostomy is a well-described adverse event.^2^^,^^3^ Laparoscopic hiatal hernia repair can also rarely result in hiatal stenosis, manifesting as postsurgical dysphagia.^4^ Herein, we describe a case in which the stent-in-stent technique is used to remove a retained esophageal SEMS, which was placed to treat a benign esophageal and gastrojejunostomy narrowing.

A 41-year-old woman with a medical history significant for obesity and GERD presented with progressive dysphagia to solids. Her surgical history is notable for a vertical sleeve gastrectomy, later converted to Roux-en-Y gastric bypass, 10 years prior to presentation, as well as laparoscopic hiatal hernia repair at an outside hospital 1 week prior to presentation. On admission, an upper GI series was performed and showed retention of contrast within the gastric pouch, suggestive of obstruction at the gastrojejunostomy, a small hiatal hernia (3.2 cm), as well as a dilated esophagus (Fig. 1). Delayed images showed progression of the contrast into the small bowel beyond the jejunojejunostomy anastomosis. An initial upper endoscopy was performed and showed a 2-cm hiatal hernia with mild narrowing and angulation at the diaphragmatic pinch (Fig. 8). Fluoroscopy showed a dilated esophagus and hold-up of contrast at the distal esophagus and gastrojejunostomy (Fig. 2). A 15-cm × 18-mm fully covered SEMS (Wallflex, Boston Scientific, Marlborough, Mass) was placed with distal flange in the roux limb and proximal flange secured in the distal esophagus with 2 interrupted sutures (Fig. 3). There were no mucosal abnormalities seen in the distal esophagus on this EGD. A subsequent upper GI series showed the fully covered SEMS in a stable position without hold up of contrast (Fig. 4). The patient was discharged on a clear liquid diet.

**Figure 1:**
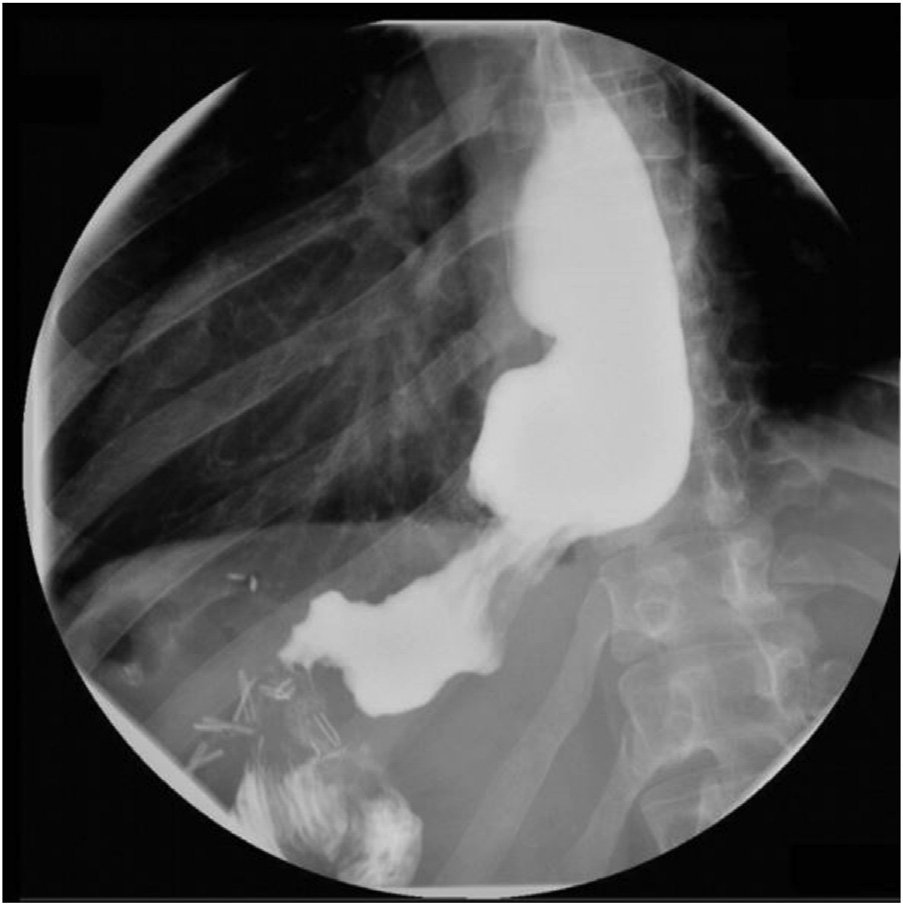
Upper GI series showing hold-up of contrast at the gastrojejunostomy anastomosis and gastroesophageal junction.

**Figure 8:**
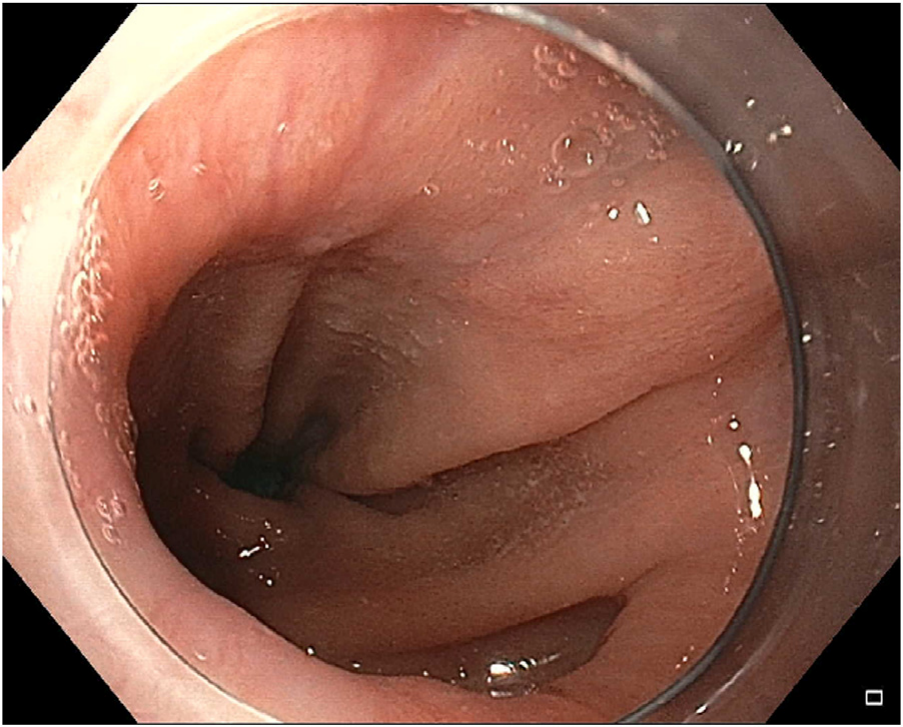
Endoscopic appearance of the gastroesophageal junction and gastrojejunal anastomosis on index endoscopy.

**Figure 2:**
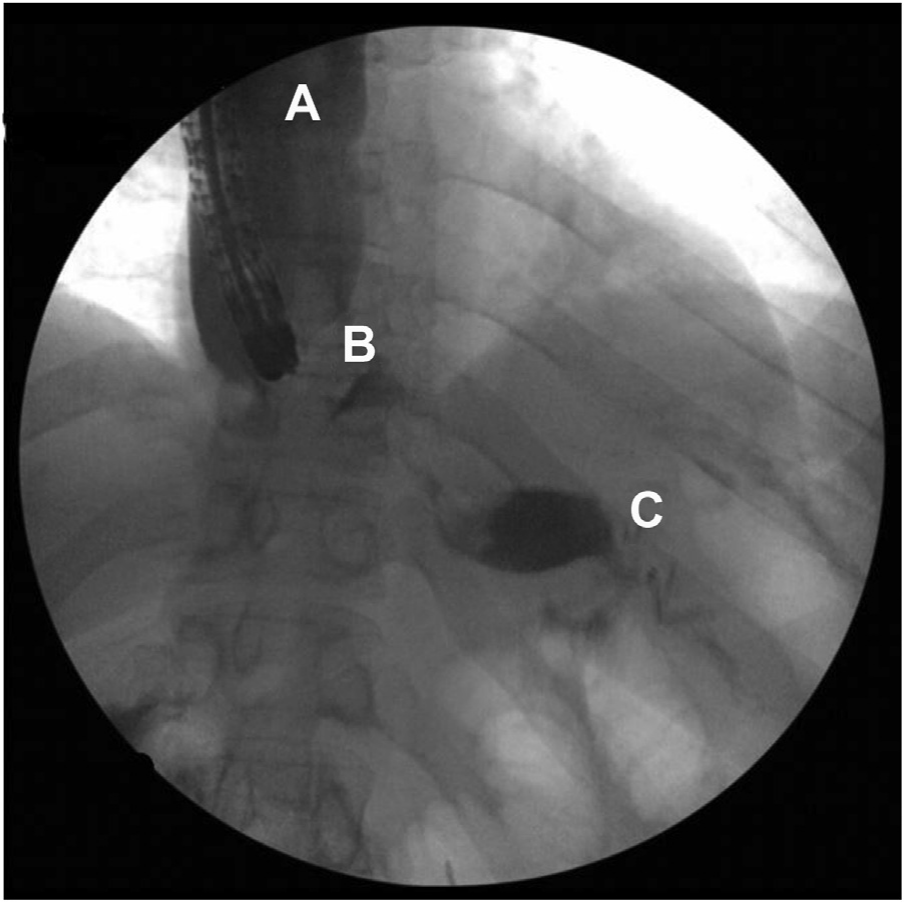
Fluoroscopy showing dilated esophagus **(A)**, hold-up of contrast at the gastroesophageal junction **(B)**, and hold-up of contrast at gastrojejunostomy anastomosis **(C)**.

**Figure 3:**
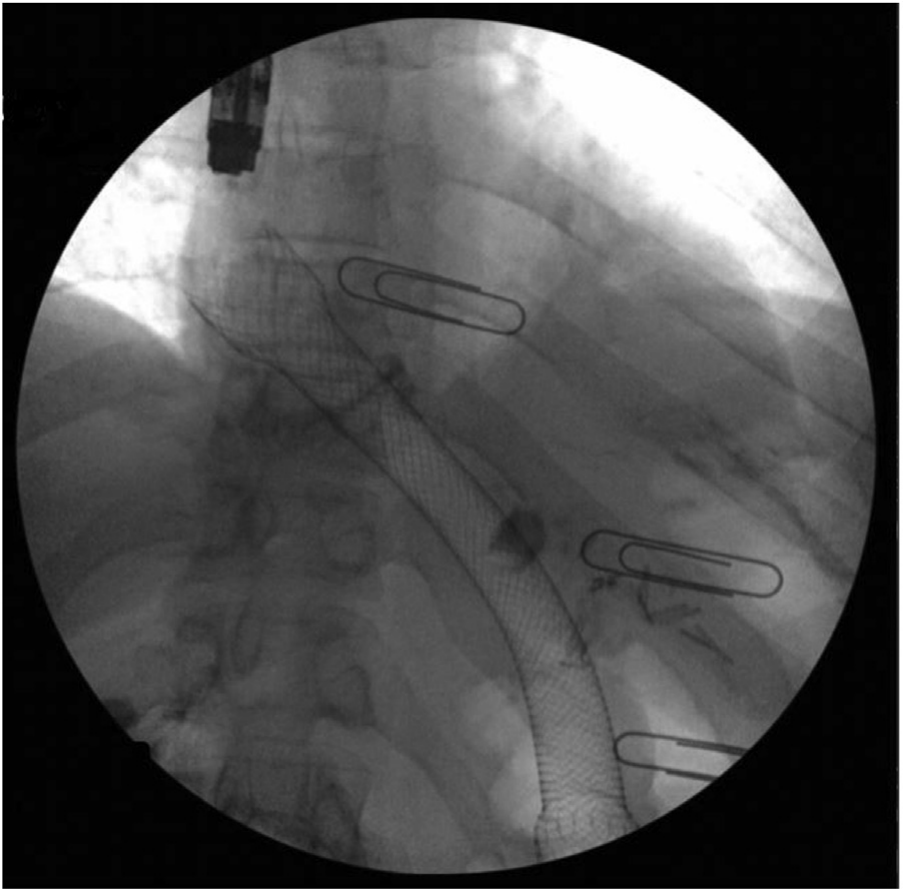
Radiograph showing the initial esophageal self-expanding metal stent remains in adequate position.

**Figure 4:**
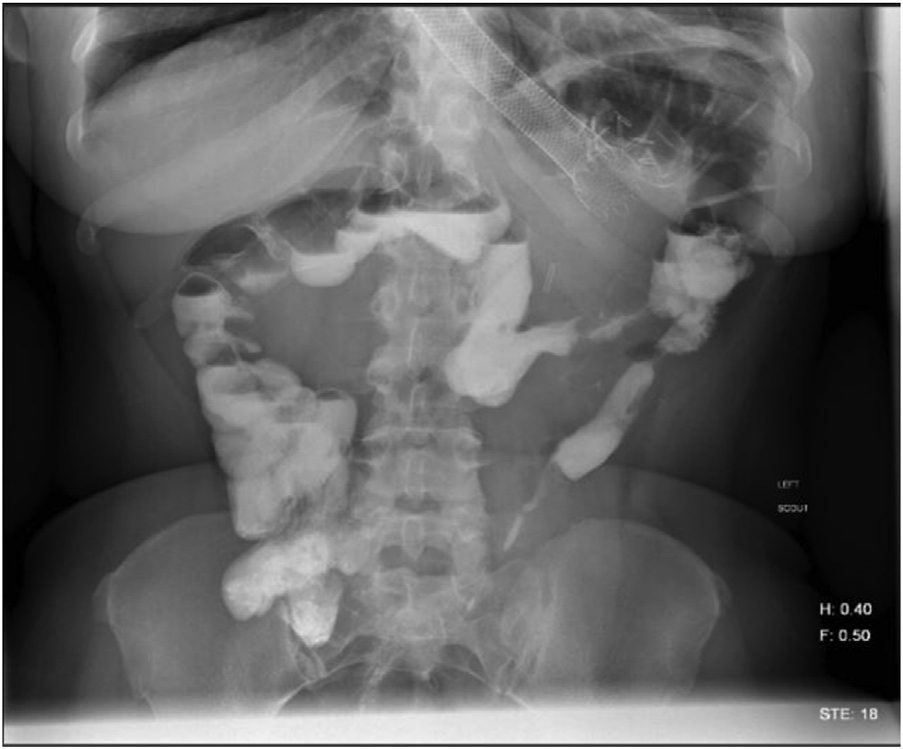
Upper GI series showing patent self-expanding metal stent.

Four weeks later, she developed recurrent nausea and vomiting. An upper endoscopy was performed and showed the SEMS in a stable location without frank obstruction. However, ingrowth of granulation tissue at the proximal flange obscured access to the sutures placed on the initial upper endoscopy. Attempts to mobilize the stent using forceps and endoscopic manipulation to improve access to the sutures were unsuccessful, and this ultimately prevented the removal of the SEMS. A second 15-cm × 18-mm fully covered SEMS was placed within the existing SEMS, with the proximal flange covering the granulation tissue ingrowth in the initial SEMS (Fig. 5). Fluoroscopy showed contrast passage through both SEMSs into the roux limb. She was then discharged on a clear liquid diet.

**Figure 5:**
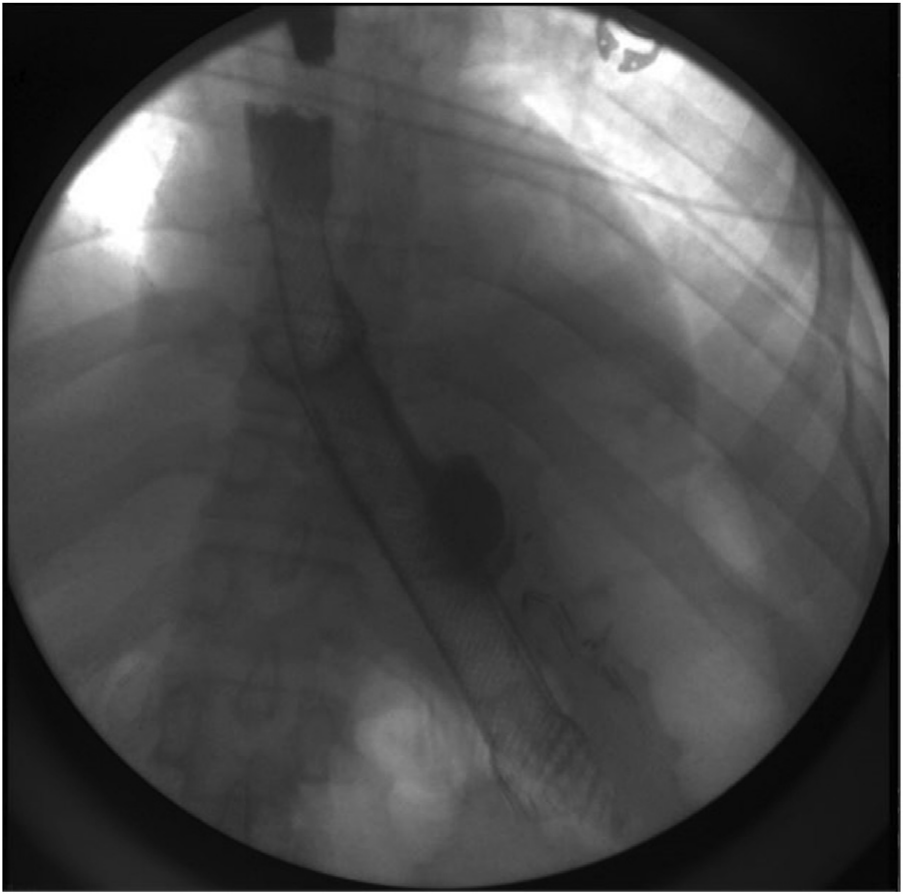
A second self-expanding metal stent placed within the initial self-expanding metal stent.

Two weeks later she returned for a planned EGD, which showed no evidence of obstruction in either SEMS or at the gastrojejunostomy anastomosis. The second SEMS was endoscopically removed and revealed a decrease in size of tissue ingrowth at the proximal flange of the initial SEMS. The remaining suture was able to be accessed and cut endoscopically, facilitating the successful removal of the initial SEMS. The residual granulation tissue in the esophagus (Fig. 6) was treated with argon plasma coagulation. Repeat endoscopy in 8 weeks showed significant reduction in the granulation tissue nodule (Fig. 7).

**Figure 6:**
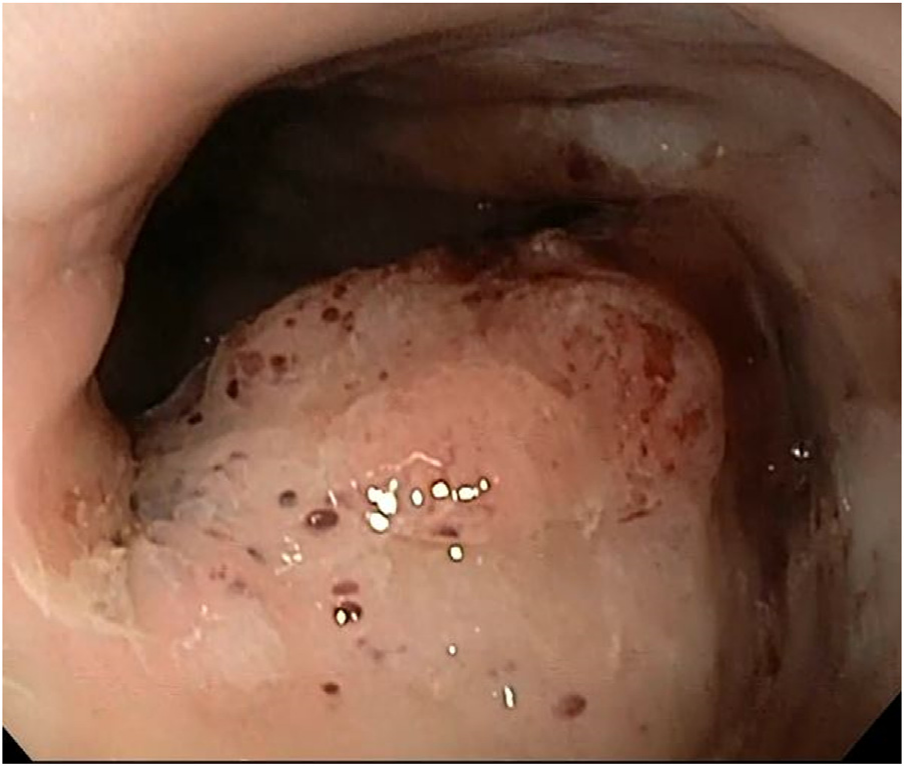
Residual granulation tissue present after the removal of both self-expanding metal stents.

**Figure 7:**
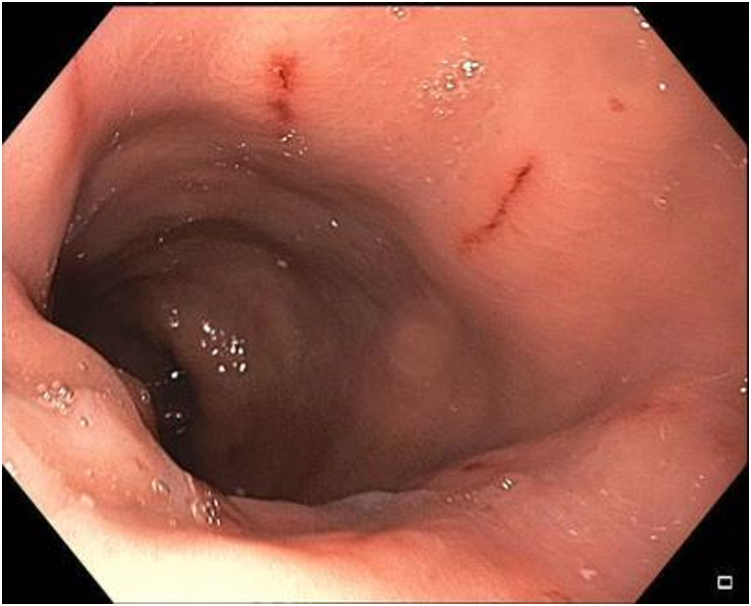
Resolution of granulation tissue after treatment with argon plasma coagulation.

Endoscopic placement of SEMSs remains preferable to plastic self-expanding plastic stents (SEPSs), largely because of higher rates of migration with SEPSs. The stent material and radial forces are thought to underly the generation of granulation tissue around the SEMS, which can serve as an anchoring force.^5^ Few small case series have demonstrated successful removal of partially or totally embedded esophageal stents using the stent-in-stent technique.^5^, ^6^, ^7^ It is hypothesized that the additional radial forces generated by the placement of a second stent induce pressure necrosis of the highly vascular granulation tissue, resulting in improved mobility of the retained stent.^5^ Furthermore, the degree of overlap between the 2 stents, implantation time, and stent material are hypothesized to also mediate the effectiveness of the stent-in-stent technique.^5^^,^^7^ As displayed in the video (Video 1, available online at www.giejournal.org), granulation tissue growth at the uncovered portion of a SEMS is a common occurrence, and the stent-in-stent technique is a safe and effective way to facilitate mobilization and extraction of a retained esophageal SEMS.

## Disclosure

*All authors disclosed no financial relationships.*

## Supplementary data

Video 1 Stent-in-stent technique for removal of retained esophageal self-expanding metal stent.
